# Supercritical Water as Nanomedium for Gasification of Lignite-Water Suspension

**DOI:** 10.1186/s11671-016-1458-x

**Published:** 2016-05-18

**Authors:** Raisa Korzh, Valerii Bortyshevskyi

**Affiliations:** Institute of Bioorganic Chemistry and Petrochemistry of National Academy of Sciences of Ukraine, 1, Murmanska str., 02094 Kyiv, Ukraine

**Keywords:** Supercritical fluids, Nanosized clusters, Ionic associates, Water-coal suspension gasification

## Abstract

The gasification of an aqueous suspension of lignite from Alexandria coalfield (Ukraine) under the supercritical pressure was studied. The initial rates of the formation of hydrogen, carbon dioxide and methane were evaluated. The mutually stimulating interaction of the components of “brown coal-water-mineral matter” system was shown due to the influence of nanoscaled water medium on the formation of dipole-inductive, dispersive and ionic associates. In the temperature range of 300–450 °C, the oxygen source for gaseous products of the lignite supercritical gasification is mainly ion-associative nanoclustered water. The source of hydrogen at the subcritical temperature is the organic part of brown coal. For the supercritical water, the source of H is the nanoscale medium with ion associates. The last ones were responsible for the further transformation of coal.

## Background

The traditional processes of steam gasification of raw coal response by the general scheme:

1 $$ {C}_x{H}_y{S}_n{N}_m{O}_z+{H}_2O\to {H}_2+CO+C{O}_2+\dots $$

**Fig. 1:**
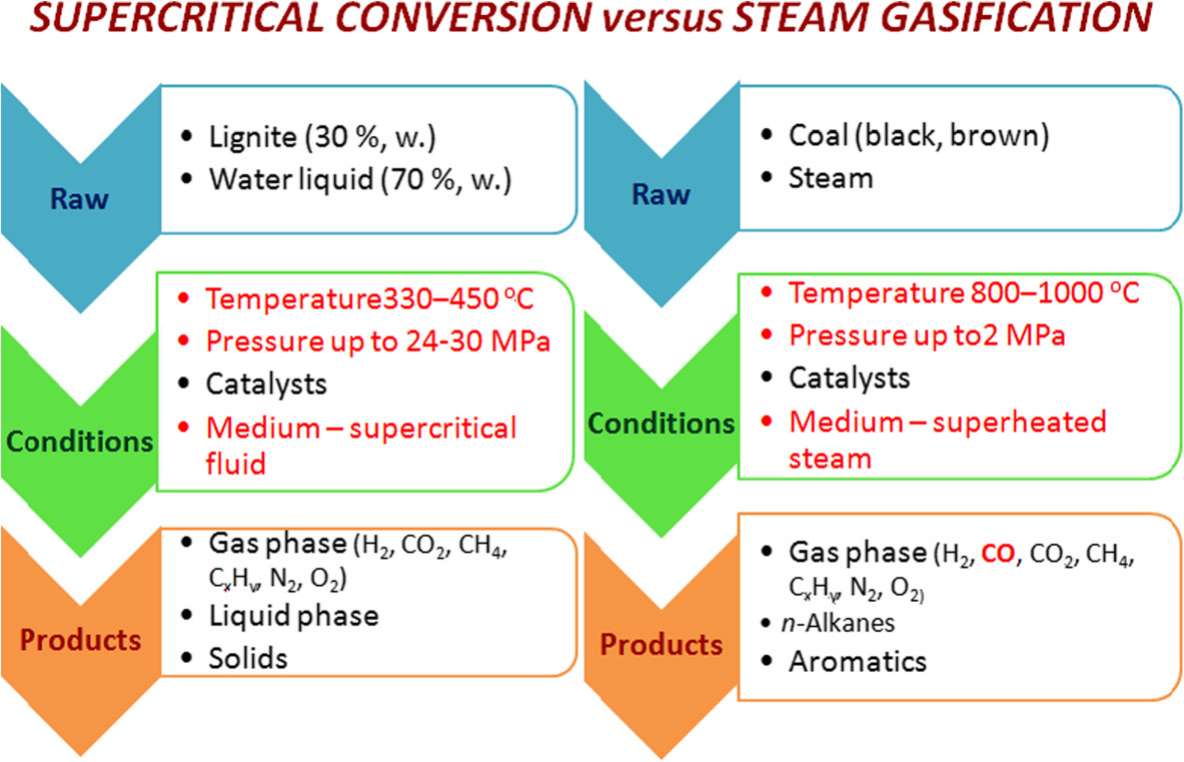
The comparison of the supercritical and steam gasification of coal

The gasification of the aqueous suspensions of coal feedstock under the supercritical water conditions is dramatically different from the steam gasification (Fig. 1). Thus, the temperature of the supercritical conversion is twice lower than the corresponding figures for the steam process and the pressure is the higher in order. Nothing differs more from the superheated steam as a reaction medium than supercritical fluid, although represented by the same agent—water.

**Fig. 2:**
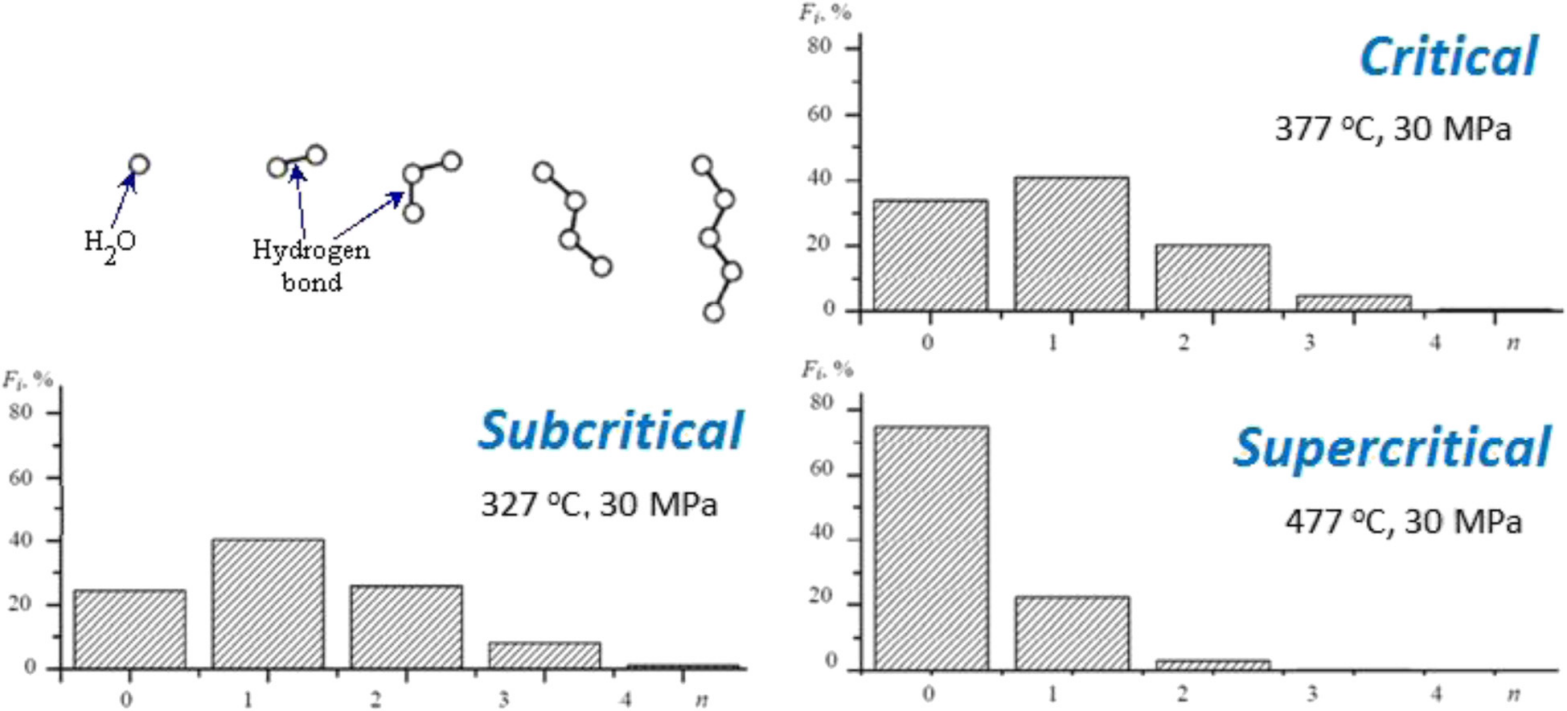
The distribution of hydrogen bonds of the supercritical water (by [2])

When water is heated above 170 °C, the net of hydrogen bonds of 150–200 molecules is destroyed and formed a “flickering” system of water clusters with from one to five molecules of water [1, 2]. It was shown by the method of molecular dynamics that in the subcritical temperature region, three molecule clusters dominate over the unbound molecules (Fig. 2). The last ones are dominated in the supercritical region of water state (up to 75 %). In turn, this transformation of the reactive medium provides changing of the distribution of formed products. The synthesis gas is dominated product of steam gasification. We have shown that the products of the supercritical gasification of lignite have no carbon monoxide within detection up to a temperature of 500 °C (see Fig. 1).

These changes of conditions, medium, and products of the supercritical gasification are caused by the changes of the mechanism of coal converting. The supercritical water gasification of coal is reviewed in [3–6]. The aim of our work was to clear the general scheme of the mechanism gasification of coal water slurry under the supercritical conditions by water. The primary problem was faced while assessing the impact of supercritical water properties on the transformation of organic matter of lignite.

## Methods

The catalytic gasification of coal-water slurry was studied under the supercritical water conditions at the facility presented in schematic diagram in Table 1. There are summarized the technological conditions of the research. More details are in [7, 8]. The main evaluated parameter was the initial rate of the formation of three major gaseous products such as hydrogen, carbon dioxide and methane.

**Table 1:** The conditions of the supercritical gasification of lignite aqueous suspension

Parameter		Supercritical gasification [7, 8]	Facility scheme
Raw		Lignite (30 %, mass)	
		Water (70 %, mass)	
Conditions	Temperature, °С	330–450	
	Pressure, MPа	24–30	
	Catalysts	NaOH, Ca(OH)_2_, NiO-MoO_3_-Al_2_O_3_ (ANM)	
	Medium	Supercritical fluid	
Products	Gaseous phase	Н_2_, СО_2_,СН_4_, С_*х*_Н_*y*_, N_2_, О_2_	1—feedstock reservoirs; 2—level meter; 3—high pressure pump; 4—back valve; 5—manometers; 6—reactor; 7—condenser; 8—pressure regulator; 9—separator; 10—gas meter; 11—reservoir for liquid and solids
	Other products	Liquids: organics + water soluble compounds (oxygenates and inorganics)	
		Solids	

## Results and Discussion

### Supercritical Gasification

The dry lignite of Alexandria deposit (Ukraine) has the general formula *С*_3.785_*Н*_2.175_*S*_0.112_*N*_0.051_*O*_1.151_. When it is the part of the aqueous suspension, it starts to gasify under the pressure of 24 MPa without the addition of catalysts at 260 °C (Table 2). The process of the lignite aqueous suspension gasification involves its interaction with water under the simplified scheme:

**Table 2:** The experimental results of the gasification of lignite aqueous suspension under the supercritical conditions

Catalyst	*Т*, °С	Initial rate of gas formation, mg/h		
		СО_2_	СН_4_	Н_2_
Without catalyst	270	37.34	0.338	0.0376
	290	35.73	0.575	0.044
	330	76.47	2.7	0.214
	450	66.1	9.89	4.3
NiO-MoO_3_-Al_2_O_3_ (AMN 10 % to coal)	330	78.06	2.856	0.38
	390	30.57	6.695	1.6
	450	15.416	18.843	3.1
NaOH (5 % to coal)	330	16.19	0.828	0.172
	390	56.44	3.017	2.17
	450	68.07	26.663	4.905
Ca(OH)_2_ (10 % to coal)	330	41.986	2.742	1.006
	390	66.873	8.975	2.153
	450	201.56	95.727	20.442

2 $$ {C}_{3.785}{H}_{2.175}{S}_{0.112}{N}_{0.051}{O}_{1.151}+{H}_2O\to {H}_2+C{H}_4+C{O}_2+\dots $$

The resulting product is divided into three phases: gas, liquid and sludge [7]. The main gaseous products are hydrogen (volume fraction of 30–50 %), methane (15–20 %), carbon dioxide (40–60 %), nitrogen (up to 5 %) and light hydrocarbons (up to 5 %). The gasification is accelerated in the temperature range of 300–350 °C, which is considered as subcritical according to [1]. Notable rate of formation of carbon dioxide, methane and hydrogen can be achieved under the heating to the supercritical temperatures above 400 °C. The addition to the reaction mixture of mineral acid catalysts and alkaline nature increases the rate of formation of hydrogen, methane and carbon dioxide with maximum values for calcium hydroxide (Table 2).

### Origin of Elements

**Fig. 3:**
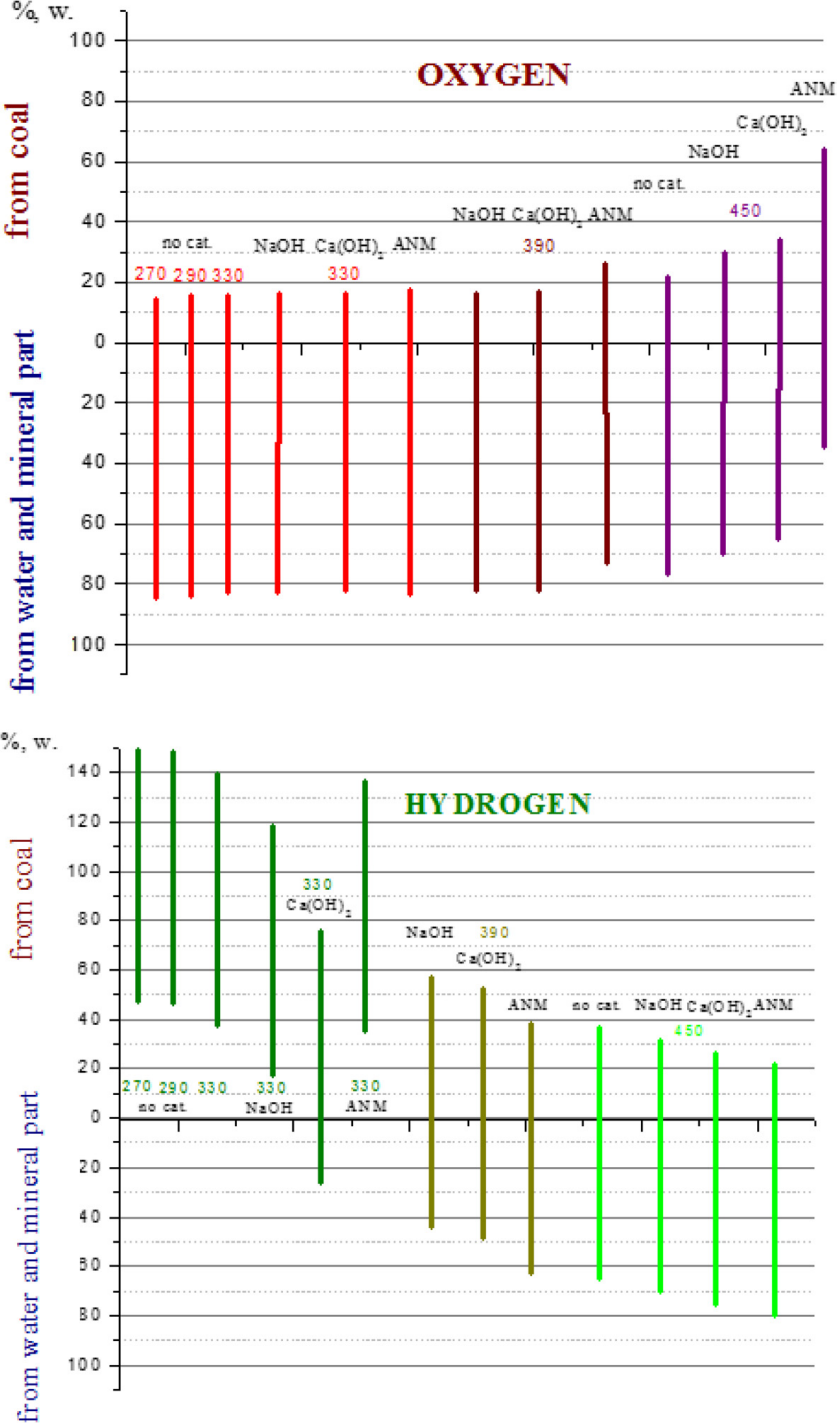
The origination of oxygen and hydrogen in gaseous products of the supercritical gasification of lignite aqueous suspension

The research technique for determining of the origination of oxygen and hydrogen is based on synchronous analysis of the CO_2_, H_2_ and CH_4_ distribution and carbon conversion at the initial rates of the gases formation. The ground is the assumption of proportional transformation of C, H and O of raw coal. Oxygen balance calculation (Fig. 3) indicates that for the subcritical temperatures below 370 °C, about 83 % oxygen passes in the gas phase from water or mineral part and only 17 % from coal. At the supercritical temperature, oxygen of inorganic origin is reduced to 63 %, but oxygen of coal origin is increased to 37 %.

According to the calculations, the own hydrogen of coal is ample for the formation all the hydrogen of gas phase at the subcritical temperature from the organic mass of coal. At the supercritical temperature, the own hydrogen of converted coal is not enough to form hydrogen of gas phase. So 70 % of the gaseous hydrogen produces from water and mineral parts.

Combining the two graphs from Fig. 3 shows the trend of the system of “brown coal-water-mineral substance” to the inversion. In the first approximation, the carbon of lignite at the subcritical temperature behaves like an acid and water is like a base; under the supercritical temperature, they switch their roles.

### The Influence of Reactive Medium

Special attention attracts the influence of the supercritical water medium and the mineral part of lignite to the coal conversion. The indicative result was no transformation of pure carbon materials like graphite by the supercritical water at temperatures ranging from 300 to 500 °C. Only when the organic, mineral and water components are combined at the supercritical pressure, the carbon is gasified. It allows us to predict the effect of the mutual stimulation (or inter amplification) of reagents in the supercritical conversion.

**Fig. 4:**
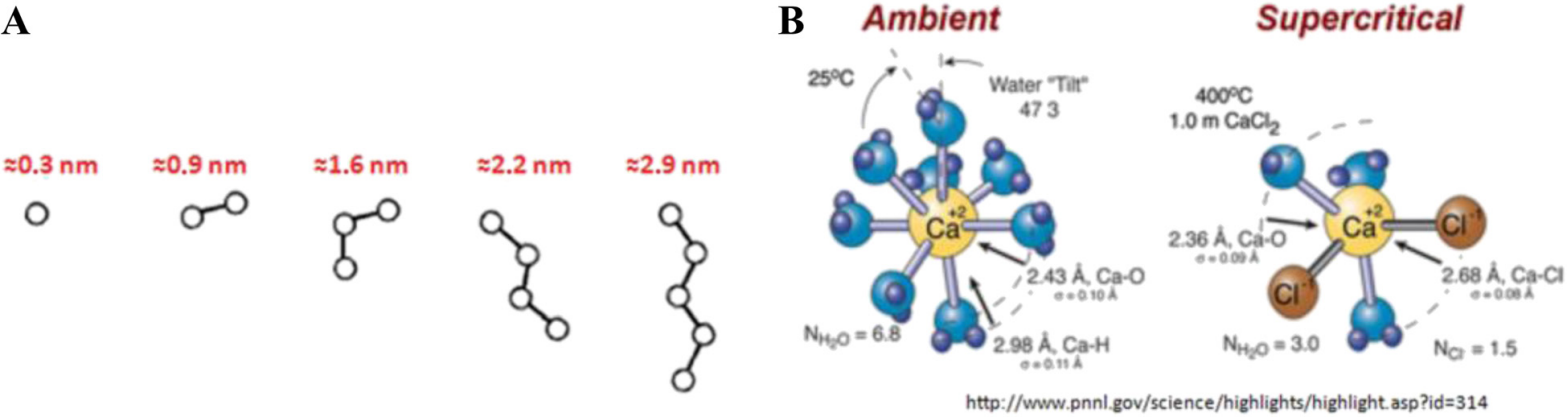
**а** Nanosizes of the supercritical water clusters. **b** The contact-ion pair of CaCl_2_ by XAFS. Adopted from [9]

It was abovementioned that the structure of the supercritical water fluid is a dynamic net of clusters from one to five molecules. Our rough calculation estimates that the size of water clusters vary in the range of 1–3 nm (Fig. 4a). Taking into account that the water component in reactor is in “flickering” regime, it is the state—one of their possible superposition states systems, description of which more correctly to conduct from position of probabilistic descriptions. Statistical approximation does not reflect the essence of occurring processes, but can give the common statistical picture as a first crude approximation. The sub- and supercritical fluid could be considered as nanoscale medium for carbon conversion. Actually, it is nanoscale water that initiates coal gasification. The nanoscale clusters of the supercritical water interact with both the organic and mineral substances of lignite. Particularly, water reacts with organic matter by the formation of the dipole-inductive and dipole-dispersive associates. Water and mineral matter produce ion-inductive interactions with the formation of nanoscale ion associates (Fig. 4b, for example calcium ion-pair from [9]). In our view, the formed nanoscale associates are responsible for the further transformation of organic and mineral parts of lignite in the supercritical medium.

**Fig. 5:**
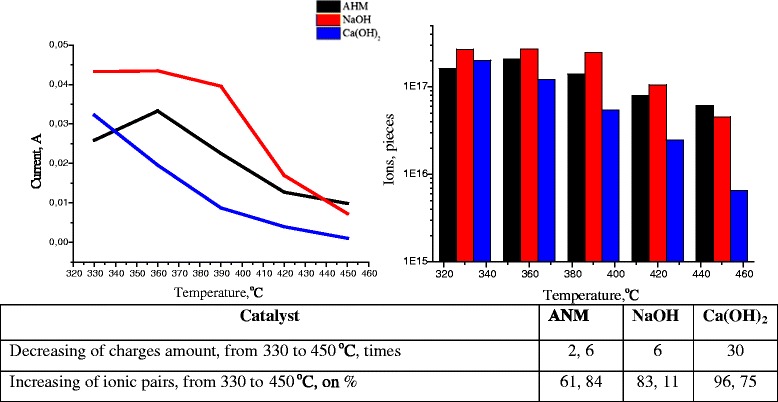
Electric conductivity of lignite aqueous suspension under the supercritical pressure while gasification processing and the calculations of number of ion pairs formed

Macroscopic display of the formation of ion pairs is falling conductivity of electrolyte solutions. We [8] experimentally showed that while the supercritical conversion under the temperature transition from 300 to 450 °C the intensity of current through lignite-water suspension decreased from 0.045 to 0.003 A (Fig. 5). Current strength was scaled to the quantity of charges. It was demonstrated that the number of charges were reduced 2.5 times for the lignite gasification in the presence of NiO-MoO_3_-Al_2_O_3_ (AMN), 6 times for NaOH and 30 times for Ca(OH)_2_. It may indirectly indicate that at 450 °C, from 60 to 96 % of carriers were transforming to the state of ion associates.

**Scheme 1:**
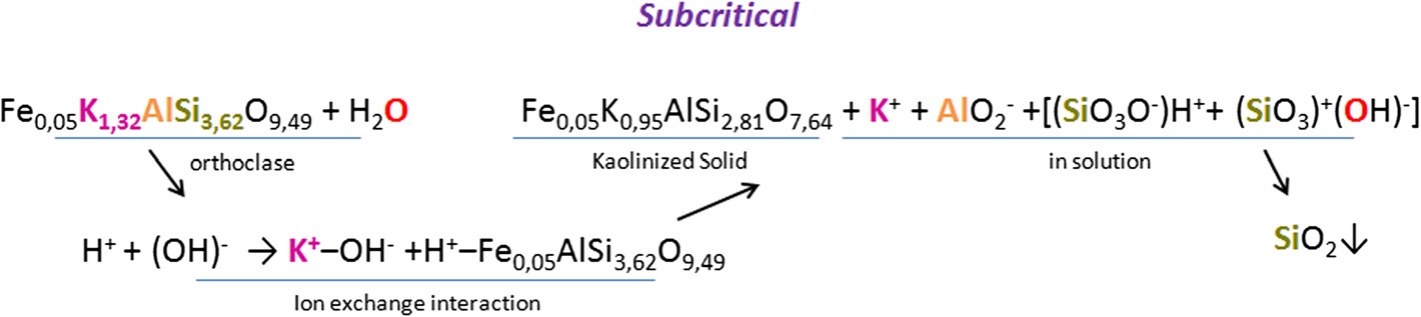
The reaction of orthoclase with the subcritical water

**Scheme 2:**
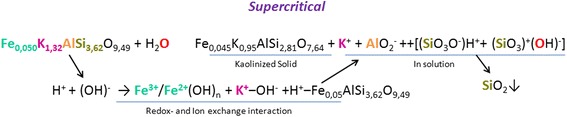
The reaction of orthoclase with the supercritical water

The process of the transformation of mineral substances in the supercritical water was separately investigated by the example of orthoclase feldspar. It was shown that orthoclase reacts with the nanoscale supercritical water with the precipitation of gray solid kaolinized phase and the saturation of the solution by products of dissociation. In particular, at the subcritical temperature, the potassium cations and aluminate anions with unstable silicon hydroxides pass from orthoclase solid into solution (Scheme 1). They form nanoscale ion associates which could accelerate the coal gasification. At the supercritical temperature, iron cations are also included to the ion exchange following the redox-transition and formation of flaky brown sediment (Scheme 2).

### General Scheme of Lignite Gasification in the Supercritical Water

**Fig. 6:**
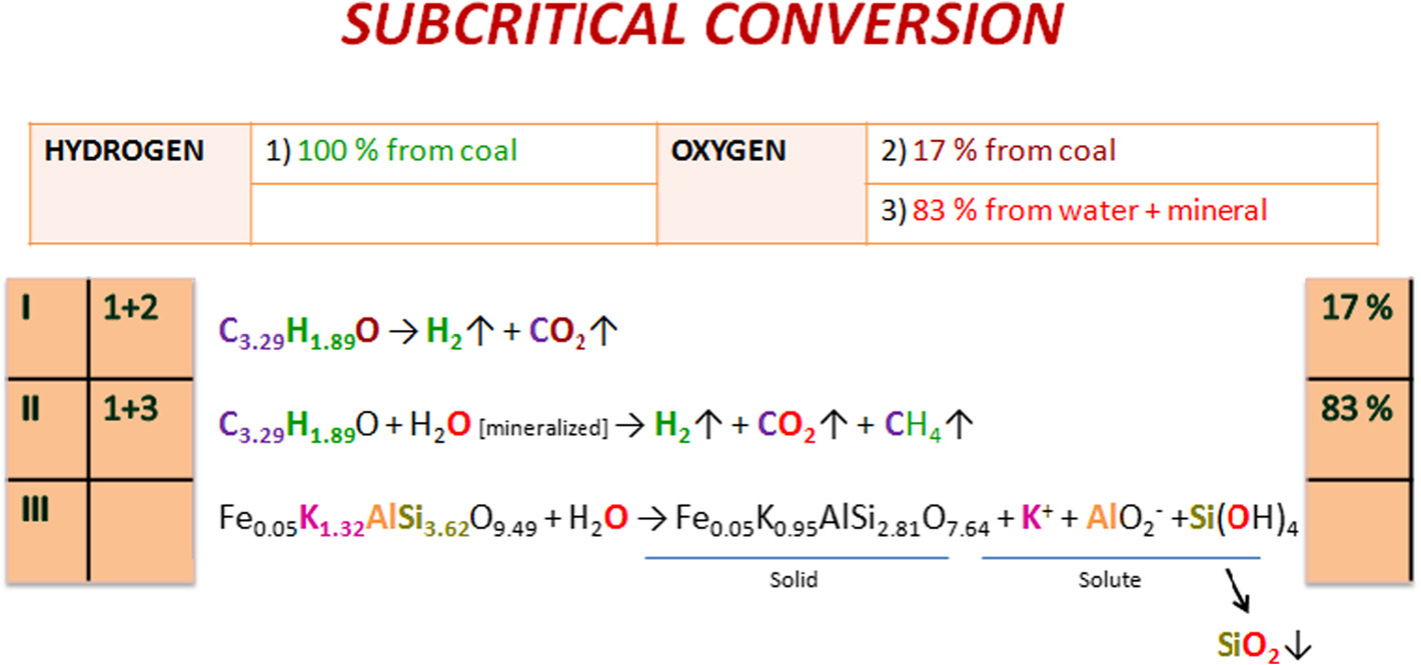
The scheme of the subcritical lignite-water slurry gasification

Compilation of data from the “Origin of Elements” and “The influence of reactive medium” sections allows proposing the general scheme of the conversion of lignite-water slurry mechanism under the supercritical pressure. Thus, there are two types of interactions at the subcritical temperatures (Fig. 6): (I) a reaction in which hydrogen comes from coal and oxygen comes from coal (1 + 2) and (II) the reaction of the involvement of hydrogen from carbon and oxygen from nanoscale mineralized water (1 + 3). The probability of their occurrence is estimated at 17/83. In the first place (or at the same time), compounds of alkali and alkaline earth metals of coal mineral part are dissolving for the formation of nanoscale ion associates by Scheme 2.

**Fig. 7:**
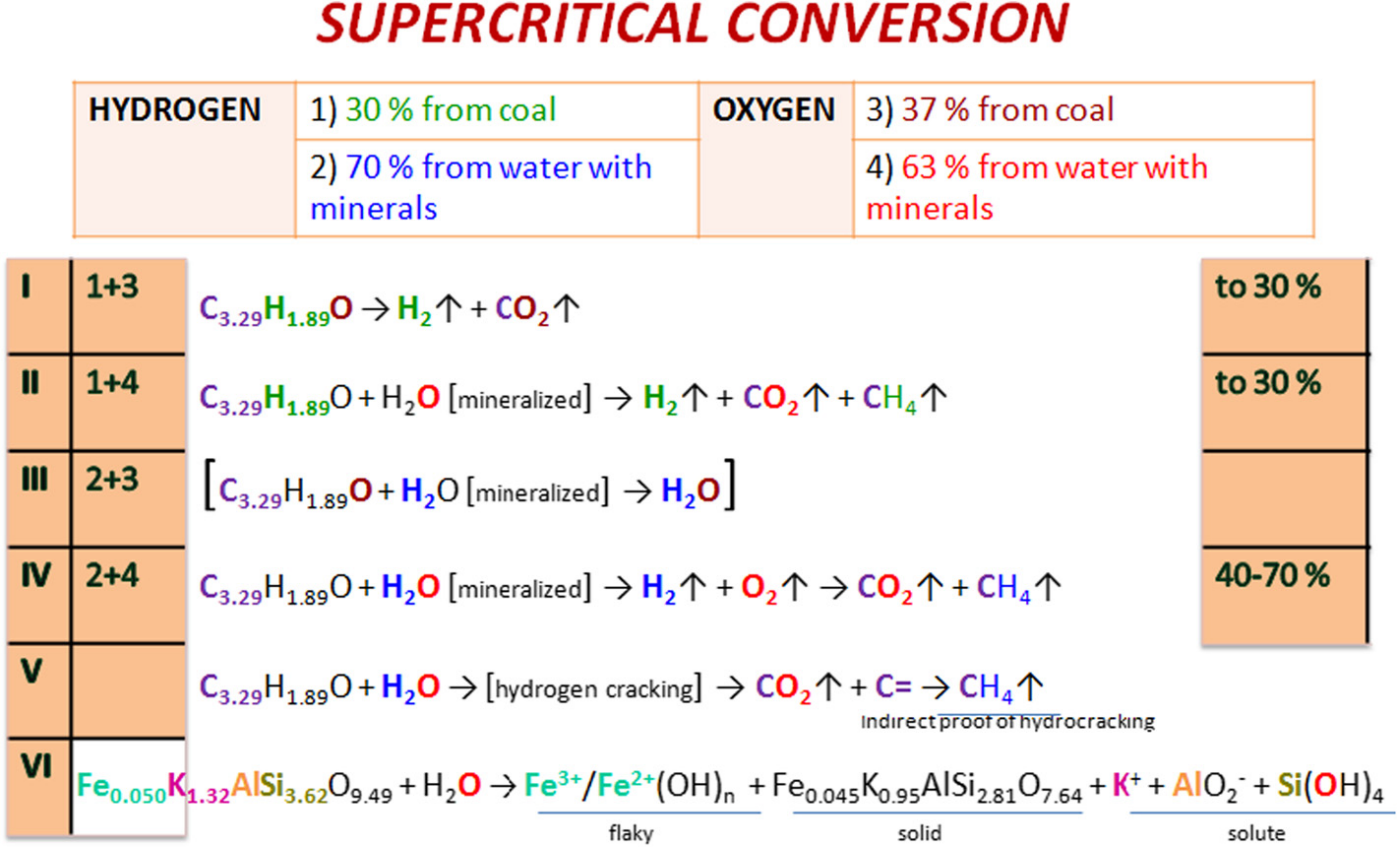
The scheme of the supercritical lignite-water slurry gasification

At the supercritical temperature, nanoscale mineralized water is input to the table of the hydrogen sources (Fig. 7). Thus, the first and second types of interactions described above are stored. The scheme is added by an interaction (III) hydrogen from nanoscale mineralized water and oxygen from coal (2 + 3) and (IV) both hydrogen and oxygen from nanoscale mineralized water (2 + 4). Interaction III leads to the formation of water, and so we take it in parentheses. Probability of I, II and IV reactions is estimated at 30, 30 and 40 %, respectively. The scheme also involved the reaction of coal hydrocracking V. The indirect evidence of the hydrocracking is the catalytic function of the aluminosilicate-based mineral substance and formed methane as a product. Ion-exchange interaction of mineral water VI is extended by dissolving and reducing of transition metal compounds.

## Conclusions

Comprehensive experimental investigation of the gasification of lignite-water slurry under the supercritical pressure shows the mutually stimulating interaction the components of the “brown coal-water-mineral matter” system due to the influence of nanoscale water medium on the formation of dipole-inductive, dispersive and ionic associates. Oxygen source for the gaseous products of the lignite supercritical conversion in the temperature range 300–450 °C is mainly ion-associative nanoclustered water. The source of hydrogen for the subcritical temperature is an organic part of lignite and for the supercritical temperature is the nanoscale medium with ion associates. Addition of acid-base catalyst accelerates the formation of hydrogen and methane. The acid catalysts under the supercritical temperature twice more attracted to convert oxygen of organic part of lignite. The alkaline catalysts enhance the transition to the gas phase of hydrogen from nanoclustered supercritical water and mineral part of lignite.
